# Multi‐omics technologies and molecular biomarkers in brain tumor‐related epilepsy

**DOI:** 10.1111/cns.14717

**Published:** 2024-04-20

**Authors:** Yaoqiang Du, Rusong Li, Danqing Fu, Biqin Zhang, Ailin Cui, Yutian Shao, Zeyu Lai, Rongrong Chen, Bingyu Chen, Zhen Wang, Wei Zhang, Lisheng Chu

**Affiliations:** ^1^ Laboratory Medicine Center, Department of Transfusion Medicine Zhejiang Provincial People's Hospital (Affiliated People's Hospital), Hangzhou Medical College Hangzhou China; ^2^ School of Basic Medical Sciences Zhejiang Chinese Medical University Hangzhou China; ^3^ The Second School of Clinical Medicine Zhejiang Chinese Medical University Hangzhou China; ^4^ Cancer Center, Department of Hematology Zhejiang Provincial People's Hospital (Affiliated People's Hospital), Hangzhou Medical College Hangzhou China; ^5^ Cancer Center, Department of Ultrasound Medicine Zhejiang Provincial People's Hospital (Affiliated People's Hospital), Hangzhou Medical College Hangzhou China; ^6^ Zhejiang BioAsia Life Science Institute Pinghu China; ^7^ School of Clinical Medicine Hangzhou Normal University Hangzhou China; ^8^ Department of Physiology Zhejiang Chinese Medical University Hangzhou China

**Keywords:** BTRE, genomics, metabolomics, molecular biomarkers, multi‐omics, proteomics, transcriptomics

## Abstract

**Background:**

Brain tumors are one of the leading causes of epilepsy, and brain tumor‐related epilepsy (BTRE) is recognized as the major cause of intractable epilepsy, resulting in huge treatment cost and burden to patients, their families, and society. Although optimal treatment regimens are available, the majority of patients with BTRE show poor resolution of symptoms. BTRE has a very complex and multifactorial etiology, which includes several influencing factors such as genetic and molecular biomarkers. Advances in multi‐omics technologies have enabled to elucidate the pathophysiological mechanisms and related biomarkers of BTRE. Here, we reviewed multi‐omics technology‐based research studies on BTRE published in the last few decades and discussed the present status, development, opportunities, challenges, and prospects in treating BTRE.

**Methods:**

First, we provided a general review of epilepsy, BTRE, and multi‐omics techniques. Next, we described the specific multi‐omics (including genomics, transcriptomics, epigenomics, proteomics, and metabolomics) techniques and related molecular biomarkers for BTRE. We then presented the associated pathogenetic mechanisms of BTRE. Finally, we discussed the development and application of novel omics techniques for diagnosing and treating BTRE.

**Results:**

Genomics studies have shown that the *BRAF* gene plays a role in BTRE development. Furthermore, the *BRAF* V600E variant was found to induce epileptogenesis in the neuronal cell lineage and tumorigenesis in the glial cell lineage. Several genomics studies have linked IDH variants with glioma‐related epilepsy, and the overproduction of D2HG is considered to play a role in neuronal excitation that leads to seizure occurrence. The high expression level of Forkhead Box O4 (FOXO4) was associated with a reduced risk of epilepsy occurrence. In transcriptomics studies, VLGR1 was noted as a biomarker of epileptic onset in patients. Several miRNAs such as miR‐128 and miRNA‐196b participate in BTRE development. miR‐128 might be negatively associated with the possibility of tumor‐related epilepsy development. The lncRNA UBE2R2‐AS1 inhibits the growth and invasion of glioma cells and promotes apoptosis. Quantitative proteomics has been used to determine dynamic changes of protein acetylation in epileptic and non‐epileptic gliomas. In another proteomics study, a high expression of AQP‐4 was detected in the brain of GBM patients with seizures. By using quantitative RT‐PCR and immunohistochemistry assay, a study revealed that patients with astrocytomas and oligoastrocytomas showed high BCL2A1 expression and poor seizure control. By performing immunohistochemistry, several studies have reported the relationship between D2HG overproduction and seizure occurrence. Ki‐67 overexpression in WHO grade II gliomas was found to be associated with poor postoperative seizure control. According to metabolomics research, the PI3K/AKT/mTOR pathway is associated with the development of glioma‐related epileptogenesis. Another metabolomics study found that SV2A, P‐gb, and CAD65/67 have the potential to function as biomarkers for BTRE.

**Conclusions:**

Based on the synthesized information, this review provided new research perspectives and insights into the early diagnosis, etiological factors, and personalized treatment of BTRE.

## Graphical abstract

Framework of identifying the multi‐omics molecular biomarkers in brain tumor‐related epilepsy (BTRE), including Genomics, Transcriptomics, Epigenomics, Proteomics, and Metabolomics, to facilitate the early diagnosis, etiological factors analysis, and personalized treatment of BTRE.

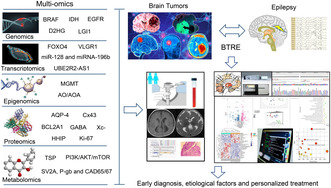

## 1 INTRODUCTION

Epilepsy is a globally prevalent neurological disorder with persistent epileptic seizures, resulting in cognitive, psychological, and social complications.^1^, ^2^ A systematic review of 222 studies (197 studies on prevalence and 48 studies on incidence) reported that the lifetime prevalence of active epilepsy was 7.60/1000 persons, and the incidence rate was 61.44/100,000 person‐years.^3^ Furthermore, individuals with lower socioeconomic status are more susceptible to developing epilepsy.^4^ Thus, epilepsy leads to severe consequences in terms of treatment cost and disease burden for both patients and their families, and seizures may result in premature patient death or may significantly affect the patient's quality of life.^5^ Repeated seizures also affect neurological development in pediatric patients, and some epilepsy patients may experience comorbidities that could further complicate their health condition.^6^

**FIGURE 1:**
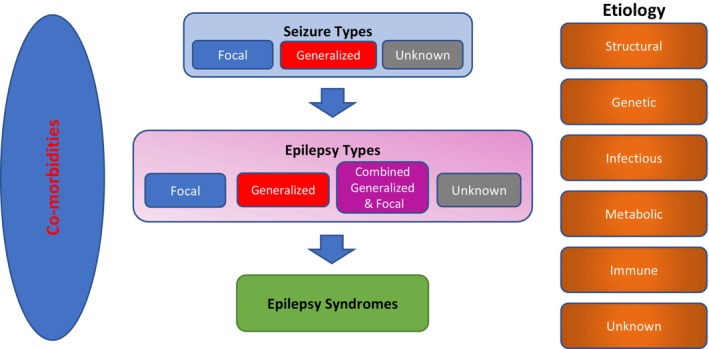
International League Against Epilepsy classification and etiologies in epilepsies.

The following three factors are considered for the early diagnosis of epilepsy: types of seizure, types of epilepsy, and epilepsy syndromes. It is also recommended to identify the underlying causes of epilepsy and its comorbidities (Figure 1).^2^, ^7^, ^8^ Despite the development of new drugs for treating epilepsy, one‐third of patients still experience seizures; therefore, epilepsy remains a worldwide critical public health concern.^9^, ^10^

Central nervous system (CNS) cancer is the seventh most common tumor in adults and constitutes 25% of malignant tumors in children; it is also the second leading cause of cancer‐related death in young people.^11^, ^12^ Brain tumors are the leading cause of epilepsy and the second most common histopathological diagnosis in the surgical specimens of patients with epilepsy.^13^ Brain tumors that originate from the brain tissue are termed primary brain tumors (such as astrocytic tumors, oligodendroglial tumors, and glioneuronal tumors). Malignant brain tumors that metastasize from other parts of the body are termed secondary brain tumors (such as lung cancer and melanoma).^14^ The incidence of primary brain tumors (including malignant and non‐malignant tumors) in the United States was reported as 24.71/100,000 inhabitants per year,^11^ which is approximately 1.6% of all cancer cases.^15^ The incidence of brain metastatic tumors was approximately 25% in individuals with solid tumors.^16^ Several studies and data sources report varying information on morbidity related to brain tumors; for example, in brain tumor‐related epilepsy (BTRE), the morbidity risk largely depends on the types of cancer. Patients with diffuse low‐grade gliomas (LGGs) patients often experience BTRE in 60%–90% (65%–90%) of cases, while those with glioblastomas (GBMs) show seizures in 60%–70% (30%–60%) of cases; moreover, patients with meningiomas experience seizures in 40%–50% of cases, and the incidence of BTRE in patients with brain metastatic tumors is 10%–20%.^13^, ^16^, ^17^, ^18^, ^19^ Because tumor‐related seizures are very persistent, most patients tend to show eventually relapse, even when treated with the best current treatment strategies. The simultaneous treatment of both tumors and epilepsy is more difficult because seizures often signal tumor progression.^18^ To enable more precise and individualized treatment of brain tumors, the first step is to achieve an accurately diagnosis of the tumors, which requires information on the basic pathological features and molecular biomarkers of these tumors.^19^

In this article, we reviewed research studies on BTRE published in the last few decades and discussed the present status, development, opportunities, challenges, and prospects in the diagnosis and treatment of BTRE based on omics studies (including genomics, transcriptomics, epigenomics, proteomics, and metabolomics).

## 2 OMICS STUDIES ON THE OCCURRENCE, DEVELOPMENT, AND MECHANISMS OF DISEASES

Multi‐omics analysis includes studies such as genomics, epigenomics, transcriptomics, proteomics, and metabolomics related to an organism or a disease. Omics technology is thought to be highly beneficial for identifying precise tumor markers associated with epilepsy, which can enhance our comprehension of the pathogenetic mechanisms of BTRE.^20^ The omics biomarkers of BTRE are crucial for the early detection of brain tumors and for selecting appropriate approaches to treat brain tumors and control epilepsy.

**FIGURE 2:**
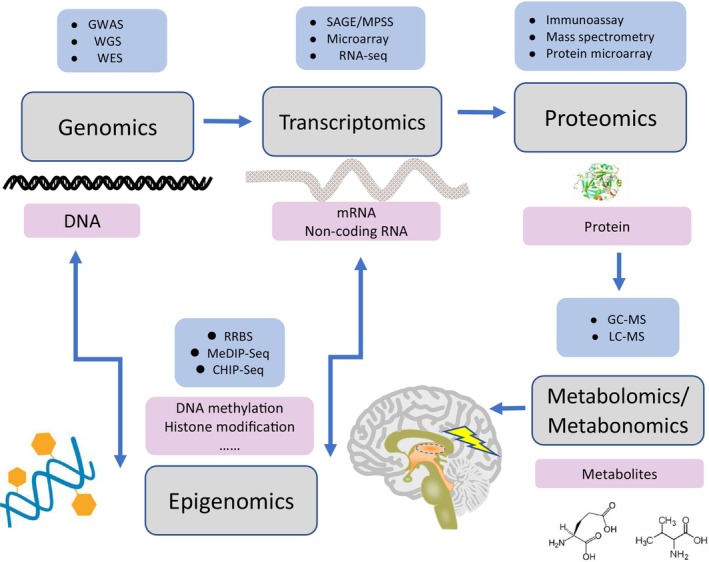
Multi‐omics studies in brain tumor‐related epilepsy, including genomics, transcriptomics, proteomics, epigenomics, and metabolomics/metabonomics studies.

During the 1990s and 2000s, the Human Genome Project was conducted, which led to the rapid development of sequencing technologies.^21^ Simultaneously, rapid advances were achieved in omics technologies and profiles analysis through the multi‐omics approach.^19^ Because BTRE is a complex and multifactorial disease, the evolving omics technologies can enable to analyze the DNA, RNA, metabolites, and proteins in the tumor and peritumoral tissues, and the rapid development of multi‐omics technologies can facilitate the investigation of the biological characteristics and molecular biomarkers of BTRE from different perspectives (Figure 2; Table S1). The application of modern sequencing computational analysis and the integration of multi‐omics approaches will further enhance and deepen the understanding of the mechanisms underlying BTRE development; this could contribute to the development of new antiepileptic or antitumor drugs and improve the life quality and prognosis of patients.^22^, ^23^

### 2.1 Genomics technologies and molecular biomarkers

Genomics involves the study of the structure and function of the complete genome of an organism; this includes all regions of the genome, such as coding, noncoding, and regulatory regions of genes.^24^ Although the Sanger dideoxy‐nucleotide sequencing method (also termed first‐generation sequencing technology) was introduced in 1977, substantial advancement in genomics was achieved in the 2000s through the use of second‐generation sequencing technology (also termed next‐generation sequencing, NGS).^25^ Some approaches known as “third‐generation sequencing technologies” have gained popularity in recent years, primarily because they do not require PCR amplification and can simultaneously scan a large number of bases.^26^ Furthermore, the rapid development of other novel technologies such as single‐cell sequencing (SNS) exponentially decreased the assay cost.^27^ Genome‐wide association study (GWAS) primarily aims to identifying single‐nucleotide polymorphisms (SNPs) in the entire genome and involves the use of microarrays for genotypic analysis of DNA samples from a large case–control cohort.^28^ Whole‐genome sequencing (WGS) primarily examines variants in the coding and noncoding regions as well as structural variants regions; however, it has some limitations such as generation of a massive amount of sequencing data, high cost, and unsuitability for identifying low‐abundance variants.^29^ In contrast, whole‐exome sequencing (WES) specifically searches for coding variants in the exon regions and is a cost‐effective alternative. WES is the most widely used approach for genomics sequencing, despite concerns regarding the loss of a large number of genes with unknown functions.^30^

V‐raf murine sarcoma viral oncogene homolog B1 (*BRAF*) encodes a serine/threonine‐specific kinase, which is one of the most important transduction factors of MAPK (RAS‐RAF‐MEK‐ERK) and has the strongest kinase activity in the RAF family. A meta‐analysis of 12 studies found that the role of *BRAF* appears in approximately 35% of BTREs.^31^ Marucci et al. detected *BRAF* variants in approximately 50% of tumor cases and demonstrated that these variants were associated with seizures in low‐grade tumors.^32^ Several studies found a statistically significant difference in the age of onset between patients harboring the *BRAF* V600E mutant and the wild‐type gene.^33^, ^34^ The associated mechanisms reflect tumor cell proliferation regulation through the RAS‐RAF‐MEK‐ERK‐MAP kinase signaling pathways, which enhanced tumor cell proliferation.^35^ Furthermore, mice experiments revealed that the *BRAF* V600E variant induces epileptogenesis in the neuronal cell lineage and tumorigenesis in the glial cell lineage.^36^ Another study indicated that *BRAF* variants can activate the mTOR signaling pathway.^37^ The other tumors developed through alterations in *KRAS*, *RAF*, *NF1*, or *FGFR1/2* genes, thus confirming that ganglioma occurs through changes in the RAS‐RAF‐MAPK pathway.^38^

The epidermal growth factor receptor (*EGFR*) gene is associated with seizure risk in glioma patients both preoperatively and postoperatively. Yang et al. used the fluorescence in situ hybridization (FISH) technique to analyze DNA obtained from 198 Chinese patients with anaplastic gliomas. Patients with preoperative seizures showed a higher *EGFR* amplification and a lower *Ki‐67* expression level.^39^ Two years later, Yang et al. conducted a similar study that included 147 high‐grade gliomas (HGGs) patients; by performing immunohistochemical staining, the authors found that patients with lower expression levels of *MGMT* and *EGFR* and those with anaplastic oligodendroglioma/anaplastic oligoastrocytoma (AO/AOA) had a higher frequency of postoperative seizure development.^40^ Isocitrate dehydrogenase (*IDH*) isoenzyme is one of the most commonly mutated genes in gliomas, and *IDH* variants occur in over 70% of grade II–III gliomas and a few cases of primary glioblastoma multiforme (GBM).^41^ Several studies have shown that *IDH1* variants result in more easy development of BTRE in preoperative,^42^, ^43^, ^44^, ^45^, ^46^ perioperative,^47^ and postoperative patients.^48^ However, some studies have shown that *IDH* variants are not related to postoperative seizure,^45^, ^49^ or are negatively associated with postoperative seizure control.^48^ A similar result for predicting preoperative seizure was found for *IDH2*.^50^, ^51^ *IDH1* variants also improve the overall prognosis of glioma patients, which may be useful for the early diagnosis of related seizures.^50^, ^52^ Furthermore, according to WHO staging, *IDH* variants are more commonly detected in LGGs.^53^, ^54^ Several studies have linked *IDH* variants with glioma‐related epilepsy, and the overproduction of D‐2‐hydroxyglutarate (D2HG) is considered to play a role in neuronal excitation that results in seizure development.^42^, ^54^, ^55^, ^56^, ^57^ Manuel Toledo et al. found that younger age, *IDH1* R132H variant, and p53 overexpression (>40%) were associated with seizures during onset.^58^

The leucine‐rich glioma‐inactivated gene 1 (*LGI1*), which encodes a protein rich in leucine and located on human chromosome 10q24, was initially identified as a potential tumor suppressor gene for glioma.^59^ This gene is downregulated in glioblastoma, thus suggesting that it might have a tumor suppressor effect.^60^ A previous study indicated that the presence of serum LGI1 autoantibodies in some HGG patients may have an epileptic effect.^61^ Additionally, *LGI1* variants have been shown to induce autosomal dominant lateral temporal lobe epilepsy (ADLTE)^62^, ^63^ and acquired autoimmune limbic encephalitis (LE),^64^, ^65^ which are associated with epilepsy. Otherwise, Huang et al. used high‐performance liquid chromatography (DHPLC) to examine the 1p and 19q status in 110 cases,^66^ their findings revealed that patients without loss of heterozygosity (LOH) on 19q were more prone to develop seizures, particularly secondary generalized seizures, than those patients with this genetic alteration.

In summary, the commonly used genomics techniques, including GWAS, WGS, WES, target‐genome sequencing, and FISH, can contribute to further explore the genomic pathogenic factors and loci of BTRE.

### 2.2 Transcriptomics technologies and molecular biomarkers

#### 2.2.1 Overview of transcriptomics technologies

Transcriptomics technology involves the analysis of gene expression and transcriptional regulation at the RNA level in cells or tissues, which includes various types of RNAs such as mRNAs, microRNAs (miRNAs), long noncoding RNAs (lncRNAs), and circular RNAs (circRNAs).^67^ Gene expression microarrays can be used to examine the overall mRNA expression and exhibit differential regulation during disease progression. This technique employs probe hybridization on microchips to detect nucleotides in samples, and it is particularly well suited to analyze mammalian transcriptomes.^68^ Microarrays, however, have limitations in analyzing low‐abundance transcripts and cannot detect point mutations, gene expression abnormalities, or methylation issues related to single‐gene inherited diseases. RNA sequencing (RNA‐seq) is a modern technology that provides comprehensive transcriptome information and identifies unknown RNA. It utilizes the capabilities of NGS to detect and quantify RNAs. Currently, RNA‐seq provides excellent resolution, high sensitivity, and bioisomer identification ability;^69^ this can enable to discover and transcript dysregulation previously overlooked by other technologies.^70^ RNA‐seq offers precise measurements of transcript levels and their isoforms, thereby allowing an impartial and comprehensive mapping of the molecular components, which is known as transcriptome profiling.^71^

Over the last two decades, various occurrences of abnormal gene expression have been documented in different types of epilepsy.^72^, ^73^, ^74^, ^75^ However, there is a relative lack of studies that utilize an expression spectrum approach to analyze the transcriptome profile of patients with BTRE. Future studies on BTRE transcriptomics should conduct mechanistic investigations aimed at elucidating the pathogenetic mechanism of BTRE.

#### 2.2.2 Role of coding RNAs in BTRE

Coding RNAs are generally mRNAs, the product of DNA transcription. Wang et al. analyzed the clinical phenotype and RNA sequence data of 86 patients with LGGs from the Chinese Glioma Genome Atlas (CGGA) database;^76^ the authors found that the high expression level of Forkhead Box O4 (*FOXO4*) was associated with a reduced risk of epilepsy occurrences. *FOXO4* was also found to be a predictor of seizure outcomes in patients with LGGs at 6 months following tumor removal. Fan et al. conducted an analysis of RNA‐seq data from 76 patients with LGGs;^77^ the authors found a significant difference in the expression level of RAD50 interactor 1 (*RINT1*), wherein patients with high *RINT1* expression were at a higher risk of developing LGG‐related seizures. The very large G‐protein‐coupled receptor‐1 (*VLGR1*) protein is the largest cell surface protein currently known and belongs to the G‐protein‐coupled receptor family. A study performed RNA‐seq of 80 patients with LGGs, and *VLGR1* was noted as a biomarker of epileptic onset in patients; furthermore, the authors found that the low expression of *VLGR1* was one of the major factors in LGGs‐associated seizures and predicted a higher incidence of seizures. *VLGR1* contains multiple Ca^2+^ exchanger β (Calx‐β) repeats with structures similar to the regulatory domains of Na^+^/Ca^2+^ exchangers; ion channels provide the basis for the regulating excitability in the CNS and are considered to be closely associated with the molecular pathogenesis of epilepsy.^78^ VLGR1‐Ca^2+^ binding is localized to the cell surface, and changes in Ca^2+^ channels are associated with the molecular pathogenesis of epilepsy.

#### 2.2.3 Role of noncoding RNAs in BTRE

Noncoding RNAs are the type of RNAs that do not encode proteins. These RNAs are transcribed from the genome but are not translated into proteins. miRNAs are noncoding RNAs that regulate gene expression in eukaryotes. They are approximately 20–25 nucleotides in length and are produced by cleavage and processing of longer primary transcripts by nuclease.^79^ lncRNAs are RNA molecules that exceed 200 base pairs in length. Unlike mRNAs, they do not encode proteins but play a crucial role in regulating gene expression at both transcriptional and translational levels through various mechanisms.^80^ miRNAs and lncRNAs are factors that control gene expression and play a role in neuron development, metabolism, and other activities.^81^, ^82^, ^83^, ^84^, ^85^ Several miRNAs, such as miR‐128^86^ and miRNA‐196b,^87^ participate in BTRE development. Yuan et al. analyzed 53 patients with LGGs and found that miR‐128 is negatively associated with the possibility of tumor‐related epilepsy development.^86^ Yang et al. also analyzed the data from The Cancer Genome Atlas (TCGA) by using similar methods and concluded that miR‐128 expression was not associated with glioma‐associated epilepsy in WHO grade 2 gliomas.^88^ The available evidence, however, cannot confirm or reject the relationship between miR‐128 and BTRE. You et al. found higher expression of miR‐196b in the tumor tissues of LGG patients with preoperative seizures.^87^ Previous studies have shown that the lncRNA UBE2R2‐AS1 inhibits the growth and invasion of glioma cells and promotes apoptosis through the miR‐877‐3p/TLR4 pathway. Xu et al. reported that UBE2R2‐AS1 expression was positively associated with overall survival in patients with glioma and functioned as a protective factor for glioma prognosis.^89^

### 2.3 Epigenomics technologies and molecular biomarkers

Epigenomics is the study of chemical modifications that occur mostly in an organism's DNA and histones. These modifications include DNA methylation and various types of histone modifications (methylation and acetylation). These mechanisms regulate gene expression by controlling chromatin composition, structure, and dynamics, thus indicating the importance of epigenetic changes in tumorigenesis.^90^

DNA methylation is a chemical modification process that involves the covalent binding of a specific base in the DNA sequence with S‐adenosyl methionine as a methyl donor.^91^ The methods used to detect DNA methylation include methylated DNA immunoprecipitation sequencing (MeDIP‐Seq), reduced representation bisulfite sequencing (RRBS), and methylation‐sensitive restriction enzyme sequencing (MRE‐Seq).^92^ Currently, the abnormal methylation of gene promoters is used as a molecular marker of tumorigenesis in oncology.^93^ Various types and sites of histone modifications exist, which can be primarily identified by chromatin immunoprecipitation sequencing (CHIP‐Seq).^94^ Although histone modification is a crucial regulatory mechanism of gene expression in cells, a limited number of studies have investigated its correlation with BTRE. High‐throughput sequencing technology can potentially reveal additional modification sites, which could help in understanding BTRE mechanisms and facilitate the identification of early diagnosis, and treatment markers through histone modification. Feyissa et al. studied 68 glioma patients by performing PCR analysis and found that patients with *MGMT* gene promoter methylation exhibited control of postoperative seizures.^48^ In contrast, another study reported that patients with lower *MGMT* and *EGFR* expression and those with AO/AOA showed more frequent episodes of postoperative seizures.^40^

Currently, there is a lack of research on the role of epigenetic biomarkers in BTRE. Following advancements in detection technology, epigenomics studies will play an essential role in identifying BTRE markers.

### 2.4 Proteomics technologies and molecular biomarkers

The traditional methods of protein separation mainly include electrophoresis such as two‐dimensional gel electrophoresis (2‐DE) and two‐dimensional difference gel electrophoresis (2D‐DIGE). In recent years, several new techniques have emerged that enable simultaneous separation and identification of proteins, such as gas chromatography (GC) or liquid chromatography (LC) coupled with mass spectrometry (MS) (GC‐MS or LC‐MS, respectively).^40^ Xu et al. performed a quantitative proteomics analysis based on the dynamic changes of protein acetylation in epileptic and non‐epileptic gliomas by using label‐free MS.^95^ The overexpression of the adenosine kinase (*ADK*) and adenosine deaminase (*ADA*) genes was found to be associated with neuronal hyperexcitability and seizure activity in temporal lobe epilepsy.^96^ Huang et al. noted no significant differences in the numbers of ADA‐ and ADK‐positive cells in the tumor tissues between glioma patients with and without seizures.^97^ This result was consistent with that reported by the European research team of de Groot et al.^98^

Aquaporin‐4 (*AQP‐4*) is the most abundant AQP (hydrophobic integral membrane proteins) family member in the brain; it is involved in the clearance of extracellular potassium and may play an important role in glutamate efflux. Isoardo et al. found a high expression of the water channel AQP‐4 in the brain of GBM patients with seizures.^99^ Although the mechanism that regulates APQ‐4 expression in GBM patients remains unclear, a post‐translational modification is suggested to be involved. Wang et al. conducted a retrospective cohort study of 186 glioma patients;^100^ based on the results of the immunochemical assay, the authors showed that glioma patients with a positive variant of the *ATRX* gene in the non‐epilepsy group were less likely to develop epilepsy than those in the epilepsy group. You et al. studied 54 patients with WHO grade II astrocytomas by quantitative RT‐PCR and immunohistochemical assay,^101^ and the authors found that patients with astrocytomas and oligoastrocytomas showed high BCL2A1 expression, which was also associated with poor seizure control. This finding suggests that *BCL2A1* is a potential biomarker in the postoperative seizure control of patients with LGGs. Cx43 is a multifunctional protein that forms hemi‐ and gap junction channels; it has several binding domains that can interact with specific Cx43‐associated proteins and plays a crucial role in many physiological and pathological processes.^102^ The *Cx43* gene is highly expressed in astrocytes under typical physiological conditions. However, Cx43 expression is reduced as the cell transforms into a malignant one. Sin et al.^103^ and Ye et al.^104^ observed a decreased Cx43 expression in the tumor center with the progression of glioma malignancy. Although this evidence indicates that *Cx43* could inhibit glioma proliferation, it also implies that the upregulation of *Cx43* expression could enhance the invasion and migration of glioma cells; thus, the overall function of Cx43 is complicated. Additionally, Cx43 or GJ/hemi‐channels play a role in promoting glioma‐associated epileptic and alteration in the tumor microenvironment (TME) by modulating the excitatory neurotransmitter glutamate.^102^ The *IDH* gene variant induces an abnormal increase of D‐2‐hydroxyvalerate (D2HG) levels. The mutant *IDH* does not convert isocitrate to ketoglutarate; instead, it converts ketoglutarate into D2HG. Another study found an association between epilepsy and the release of D2HG, a chemical structurally related to glutamate, in the TME.^105^ By performing immunohistochemistry (IHC), several studies have reported the relationship between D2HG overproduction and seizure occurrence.^42^, ^54^, ^55^

Glutamate levels were found to be elevated in tumors and adjacent tissues of epilepsy patients with HGGs, and in animal models with xenografted HGG.^56^, ^106^ The increased expression of the glutamate transporter may play an essential role in the pathogenesis of BTRE.^57^ Sontheimer et al. found that treatment with sulfasalazine, a system Xc‐specific blocker,^107^ can reduce the number of spontaneous epileptic events in glioma‐implanted mice, thus suggesting that system Xc‐mediated glutamate release in the tumor is involved in seizure production. M. S. Sears et al. found that the lack of the system Xc^−^ transporter in mice resulted in decreased epileptogenesis;^108^ further studies suggest that this might be partly due to the reduction in the level of AMPA receptor subunit GluA1. The researchers also detected excitatory amino acid transporter 2 (EAAT2) downregulation and system Xc^−^ upregulation, which indicates the key roles of these three biomarkers in BTRE.^57^, ^99^ Yuen et al. noted that glutamate concentration in glioma and peritumoral tissues was significantly higher in glioma patients with epilepsy than in glioma patients without epilepsy, and glutamate concentration in peritumoral tissues was higher than that in glioma itself; these findings were consistent with those of previous studies.^99^ Rosati et al. studied samples from 83 newly diagnosed GBM patients by performing IHC; the authors found that glutamine synthetase (GS) expression patterns in neoplastic cells were inversely correlated with the presence of epilepsy,^109^ and the GS is an astrocytic enzyme that catalyzes the conversion of glutamate and ammonia to glutamine.

Different from glutamate, γ‐aminobutyric acid (GABA) mainly participates in inhibitory signaling pathways. A study indicated that compared to areas without epilepsy surrounding the LGGs, epileptic peritumoral regions contained fewer GABA‐containing neurons.^110^ GABAergic networks play an important role in epileptogenesis.^96^ Several GABA receptor subunits were downregulated in the tissues of patients with gangliogliomas as compared to that in the control group, which could lead to impaired GABAergic tonic inhibition and the subsequent disruption of excitatory–inhibitory connection in the CNS.^111^, ^112^ The inhibitory effect of GABA is mainly dependent on the K^+^, Cl^−^ cotransporter (KCC2), which extrudes Cl^−^ from the cell to maintain GABA reversal potential, while the Na^+^, K^+^, 2Cl^−^ cotransporter (NKCC1) transports Cl^−^ into the cell to increase the excitatory capability. Elevated NKCC1 expression and decreased KCC2 expression were observed in glioma tumors excised from individuals with medically intractable epilepsy^112^, ^113^; this finding was further validated by in vitro experiments conducted by Conti et al.^114^ Additionally, the alteration in the expression levels of these two transporter proteins in the neocortex around the tumor leads to the excitatory effect of GABA and induces an imbalance between synaptic excitation and inhibition during epilepsy in glioma patients.^114^, ^115^ Lin et al. studied five human synapse‐associated proteins (SAPs) and found that gamma‐aminobutyric acid type A receptor subunit delta (GABRD)^116^ was upregulated in the glioma tissue of patients with glioma‐associated epilepsy as compared to that in non‐epileptic patients; this finding indicated that SAPs may be involved in the pathogenesis of seizures in glioma patients.

The hedgehog‐interacting protein (*HHIP*) gene encodes a membrane glycoprotein and functions as an endogenous antagonist in the hedgehog signaling pathway. Chang et al. studied 135 samples of GBM patients and healthy people by IHC.^117^ Their results showed that *HHIP* expression was significantly correlated with seizure. High HHIP expression could be a protective factor, which predicts a low risk for seizure. Previous studies have shown that the inwardly rectifying potassium channel 4.1 (*Kir4.1*, also known as *Kcnj10*) plays a key role in potassium hemostasis, which is crucial in maintaining normal neural excitability.^118^ Zurolo et al. observed the downregulation of Kir4.1 expression in status epilepticus of a rat model of temporal lobe epilepsy^119^; moreover, the expression level of kir4.1 in the surgical specimens of epileptic patients with a brain tumor was also lower than that in the specimens of non‐epileptic patients, which was consistent with the findings of previous studies. The authors also found that the downregulation of Kir4.1 expression corresponded with the prominent upregulation of IL‐1β mRNA, thus suggesting the critical role of these biomarkers in tumor‐related epilepsy. *Ki‐67* is an excellent marker for determining the growth fraction of a particular cell population, and its expression level is regularly monitored in many neurosurgery centers to estimate the extent of glioma malignancy and patient prognosis. A lower Ki‐67 expression level was detected in patients with preoperative seizures.^39^ Yang Yuan et al. tested 93 histologically confirmed WHO grade II glioma tissues by using immunohistochemical staining and concluded that Ki‐67 overexpression in WHO grade II gliomas is associated with poor postoperative seizure control; thus, Ki‐67 is a potential molecular marker to predict poor seizure control after surgery.^120^ However, a contrasting result was obtained in a later study.^40^ In the mammalian brain, the N‐methyl d‐aspartate receptor (NMDAR) is a calcium‐permeable subtype of the ionotropic Glu receptor. Gao et al. found that the extrasynaptic NMDA receptor with 2B subunit (NR2B) is substantially phosphorylated at S1013 in neurons present in the peri‐glioma area.^121^

By performing genetic sequencing and an electrophysiology analysis (EEG), Tobochnik et al. found that the presence of H1047R,^122^ one of the four commonly observed variants (H1047R, R88Q, E542K, and G118D) in PIK3CA, the PI3K catalytic subunit, was significantly associated with worse seizure control. The synaptic vesicle protein 2A (SV2A) is the binding site for the antiepileptic drug levetiracetam.^123^ Romoli et al. studied specimens from patients enrolled in the multicenter COMPO study and found the high expression of SV2A can be a protective factor to predict the low risk of BTRE.^124^ Similar results were obtained by de Groot et al.^125^ However, an earlier study by de Groot et al. yielded the opposite result.^126^ Huang et al. analyzed the clinical data and immunochemistry results of 310 patients with supratentorial astrocytic tumor or oligodendroglial tumor.^127^ The authors concluded that topoisomerase II (TopoII) positivity is a strong low‐risk predictor for preoperative epileptic seizures.

In gliomas, the non‐vesicular secretion of glutamate through the cystine–glutamate exchanger (SLC7A11, xCT) is the primary mechanism responsible for the elevated concentration of extracellular glutamate. System XC (SXC), encoded by *SLC7A11*, is a cystine/glutamate antiporter that releases extracellular glutamate following cystine absorption.^128^ It is interesting to note that SLC7A11 expression is upregulated in gliomas and associated with seizure occurrence in both human and animal tumors.^129^, ^130^, ^131^ Robert et al. observed that in mice with SLC7A11‐expressing gliomas, the peritumoral neurons next to the gliomas had depolarized resting potentials and fired more action potentials than the SLC7A11‐negative gliomas, thus indicating a hyperexcitable condition in the former.^129^ Sørensen et al. studied the tumor samples of 229 glioma patients by immunochemistry.^132^ The authors found that the high xCT expression was significantly associated with seizures at onset, particularly in GBM patients. This finding is consistent with the results of previous studies.^56^, ^129^ Heroux et al. used sodium dodecyl sulfate‐polyacrylamide gel electrophoresis (SDS‐PAGE) fractionation followed by LC‐tandem MS to test GBM and epilepsy specimens; the upstream regulators STAT3 and SP1 were activated and CTNNα was inhibited.^133^

In summary, proteomics technology is used to identify, quantify, localize, modify, and detect interactions and functions of proteins. The determination of the special proteins in an organism is essential to accurately and completely discover the in‐depth mechanisms of BTRE.

### 2.5 Metabolomics technologies and molecular biomarkers

Metabolomics/metabonomics is a branch of omics that involves the quantitative analysis of all metabolites present in an organism to investigate their relative relationships during physiological and pathological changes.^134^ Because of limitations related to detection technology, current metabolomics focuses on metabolites with molecular weights below 1000 Da. Additionally, metabolites are similar across various biological systems; this enables the technologies used in metabolomics research to be more accessible and applicable to different fields.^135^ Metabolomics can be categorized into two types: nontarget metabolomics and target metabolomics. Nontarget metabolomics is typically used to broadly screen and identify various metabolites, whereas targeted metabolomics focuses on the analysis of specific metabolites.^136^ Metabolomics techniques primarily involve the use of nuclear magnetic resonance (NMR), HPLC, GC‐MS, and LC‐MS.

The thrombospondin (TSP) family is a key regulator of synaptogenesis. Wang et al. analyzed glioma rat models by EEG, and they found that TSP2 overexpression in tumor tissues caused an increase in spine density and excitatory synapses in the peritumoral region, resulting in hyperexcitability in the peritumoral cortical networks.^137^ The PI3K/AKT/mTOR pathway is associated with the development of glioma‐related epileptogenesis.^138^ A study indicated that PIK3CA may have different effects on peritumoral hyperexcitability depending on the particular variation implicated.^139^ *OLIG2* encodes the oligodendrocyte transcription factor 2 protein, a helix‐loop‐helix transcription factor that functions as a universal marker of diffuse gliomas.^140^ *RTN1* encodes the protein reticulon‐1 involved in the neuroendocrine secretion. Lee et al. utilized MRI scanning and RNA‐seq approaches to perform gene expression imaging and mapped the expression of genetic biomarkers in the affected brain area of glioblastoma‐associated epileptic seizures.^141^ These analyses indicated a significant correlation between BTRE and regions with a remarkably low expression of OLIG2 and RINT1. Neal et al. used the glutamate‐weighted imaging method GluCEST on 10 patients who have been diagnosed to have grade II–III diffuse glioma and further confirmed the role of glutamate in diffuse glioma biology.^142^ This finding further implicated the elevated level of peritumoral glutamate in epileptogenesis and altered tumor glutamate homeostasis in glioma aggressiveness. L. Douw et al. used magnetoencephalography to study the brains of glioma patients; the authors found that SV2A, P‐gb, and CAD65/67 have the potential to function as biomarkers for BTRE.^143^

At present, few studies have utilized metabolomics techniques to identify metabolites that are causally related to the BTRE pathogenesis, and this research field is highly worth exploring.

## 3 ASSOCIATED MECHANISMS OF EPILEPSY AND BRAIN TUMOR

Epileptogenesis, the process of epilepsy development, is a multifaceted and varied pathophysiological phenomenon. It is triggered by either a genetic variant or an epileptogenic insult, although the cause remains unknown in many patients.^144^ Currently, the primary causes of epilepsy are identified as structural, genetic, metabolic, and immune factors. However, a considerable proportion of epilepsy cases have an “unknown” cause.^2^ It is crucial to understand that the causative factors mentioned above are not solely responsible for inducing spontaneous wiring and disease progression. Before the occurrence of the first spontaneous seizure, several pathological changes occur in the brain tissue (including neurons and glia), such as reactive gliosis and blood–brain barrier compromise. These changes ultimately lead to a state of hyperexcitability, which is associated with a low seizure threshold.^145^ Although the underlying pathophysiological mechanism is not well understood, seizures may be induced by various factors.^146^ Mechanical compression, vascularization and oxygen demand imbalance in the tumor, inflammatory processes, and neurotransmitter imbalance (specifically GABA and glutamate) are the primary factors that contribute to epileptogenesis in patients with brain tumors.

Multiple lines of evidence suggest that glutamate, the primary excitatory neurotransmitter in the CNS, plays a crucial role in tumor‐associated seizures. The close association between seizures and oncogenesis may be attributed to the high levels of glutamate in the TME. This is caused by an increased expression of the cystine–glutamate transporter, which leads to the over‐activity of the glutamatergic signaling pathway. Consequently, seizures occur and contribute to the development of cancer.^129^, ^146^ Changes in glutamate neurotransmission have been strongly linked to the development in patients with brain tumors, particularly those with highly epileptogenic gliomas. Elevated levels of glutamate have been detected in both tumor and peritumor samples from epilepsy patients with HGG, and in animals implanted with xenografted HGGs.^56^, ^106^ The increased expression of glutamate transporter may play an essential role in the pathogenesis of tumor‐related epilepsy.^57^

An imbalances in neurotransmitters cause the tumor and the surrounding tissue to become epileptogenic. This implies that the peritumoral tissue is also a part of the “epileptogenic zone.” According to some researchers, epileptic activity may originate from areas surrounding the tumor that are within a distance of 1–2 mm from its border, rather than from the tumor itself.^14^, ^147^ Thus, even a complete resection of a brain tumor does not necessarily mean that the epileptogenic zone has been totally removed; this is because a relevant part of seizures in BTRE can also be generated by the surrounding tissue. Part of the dilemma of surgical treatment of tumor‐induced epilepsy stems from the following aspect: removal of the tumor alone cannot completely control epilepsy, while excessive removal of the peritumoral tissue can severely impair brain function. Several preclinical and clinical studies have shown the influence of neurotransmitter imbalance on epileptogenesis in BTRE, in particular, a decrease in inhibitory GABAergic neurotransmission and an increase in excitatory glutamatergic synaptic input.^148^ Although changes in the extracellular milieu may cause cortical irritability, this depends on the balance between excitatory transmitters, such as glutamate, and inhibitory factors, such as GABA. The inhibitory effect of GABA mainly relies on the KCC2 transporter, which extracts Cl^−^ from the cell to maintain GABA reversal potential. A previous study indicated that compared to areas without epilepsy surrounding an LGG, the epileptic peritumoral regions have fewer GABA‐containing neurons.^110^ Other studies have demonstrated that changes in chloride homeostasis in the peritumoral microenvironment may decrease GABAergic inhibition.^114^ The disruption of chloride homeostasis is reported in several epilepsy disorders, including BTRE.^115^, ^149^ KCC2, a cotransporter of potassium and chloride, is specifically mentioned as being involved in this process.^150^ KCC2 enables extracellular potassium and chloride release, and is crucial for preserving a chloride gradient across neurons. KCC2 also plays an important role in regulating GABA function by altering intracellular chloride levels to alternate between hyperpolarizing and depolarizing GABAergic signaling. Gliomas show altered KCC2 expression, and the downregulation of KCC2 expression is observed in the peritumoral tissue of mouse glioma models, which corresponds with the occurrence of spontaneous seizures.^150^, ^151^ KCC2 is also downregulated in the peritumoral epileptic brain cortex.^114^ Taken together, these findings suggest that the decreased KCC2 expression could increase intracellular chloride levels, which would then impair inhibitory GABA function, increase excitability, and lower the seizure threshold. Notably, KCC2 may also be downregulated in response to high glutamate concentration, and as such this mechanism may interact with and be compounded by disrupted glutamate homeostasis.^150^

NKCC1 is a chloride transporter implicated in epileptogenesis. NKCC1 is upregulated in human glioblastoma and is responsible for the increase in intracellular chloride levels.^115^ NKCC1 is also upregulation in ganglioglioma, a tumor invariably linked with seizures, and this coincides with KCC2 downregulation.^112^, ^113^ The NKCC1 antagonist bumetanide lowers seizure frequency in patients with temporal lobe epilepsy.^152^ Taken together, these findings indicate that NKCC1 is a viable therapeutic target for BTRE and necessitates more research on its role in the disease.^153^ Additional molecules involved in ion transport and considered to play a role in epileptogenesis include AQP‐4 and Kir4.1. AQP‐4 is overexpressed in GBM patients presenting with seizures, while mutations in Kir4.1 are associated with epilepsy in humans and mice models.^99^, ^119^, ^154^, ^155^

Presently, many data retrieval systems (tools and databases) extract a wide range and types of comprehensive data from various resources, including genes, proteins, metabolites, and explore the sequences, structure, function, and classification information. Databases such as GEO, TCGA, GeneCards, OMIM, ENCODE, Oncomine, and STRIDE are commonly used in research on BTRE (Table 1; Table S1). In‐depth mining and improvement of these resources, as well as their effective coupling with omics technology, will be extremely beneficial to study BTRE biomarkers and the mechanisms associated with epilepsy development.

**TABLE 1:** Multi‐omics databases and tools commonly used in related studies.

Omics	Items	Website	Description
Genomics, Transcriptomics, Epigenomics, Multi‐omics	GEO	https://www.ncbi.nlm.nih.gov/geo/	A public functional genomics data repository supporting MIAME‐compliant data submissions
Genomics, Transcriptomics, Epigenomics, Multi‐omics	TCGA	https://portal.gdc.cancer.gov/	Harmonized cancer datasets, genomic data commons data portal
Genomics, Transcriptomics, Epigenomics, Multi‐omics	ICGC	https://dcc.icgc.org/	It provides many tools for visualizing, querying, and downloading cancer data
Genomics, Transcriptomics, Epigenomics, Multi‐omics	GTEx	https://www.gtexportal.org/	It is an ongoing effort to build a comprehensive public resource to study normal tissue‐specific gene expression
Genomics, Transcriptomics, Epigenomics	CGGA	http://www.cgga.org.cn/	It contains clinical and sequencing data of over 2000 brain tumor samples from Chinese cohorts, and is equipped with a user‐friendly web application for data storage and exploration
Transcriptomics	BrainSpan	http://www.brainspan.org/static/home	The BrainSpan atlas is a foundational resource for studying transcriptional mechanisms involved in human brain development
Transcriptomics	GlioVis	http://gliovis.bioinfo.cnio.es/	A user‐friendly web application for data visualization and analysis to explore brain tumor expression datasets
Genomics, Transcriptomics, Epigenomics, Proteomics, Multi‐omics	UCSC	http://genome.ucsc.edu/	University of California, SANTA CRUZ genomics institute, UCSC Genome Browser
Genomics, Transcriptomics, Epigenomics, Multi‐omics	Ensembl	https://www.ensembl.org/index.html	It is a genome browser for vertebrate genomes that supports research in comparative genomics, evolution, sequence variation, and transcriptional regulation
Genomics, Transcriptomics	BioGPS	http://biogps.org/#goto=welcome	A free extensible and customizable gene annotation portal, a complete resource for learning about gene and protein function
Genomics, Multi‐omics	GeneCards	https://www.genecards.org/	It is a searchable, integrative database that provides comprehensive, user‐friendly information on all annotated and predicted human genes
Genomics, Multi‐omics	Gene ATLAS	http://geneatlas.roslin.ed.ac.uk/	A large database of associations between hundreds of traits and millions of variants using the UK Biobank cohort
Genomics, Transcriptomics	SMD	https://smd.stanford.edu/	It stores raw and normalized data from microarray experiments, and provides web interfaces for researchers to retrieve, analyze, and visualize their data
Genomics	OMIM	https://omim.org/	An Online Catalog of Human Genes and Genetic Disorders
Genomics, Transcriptomics, Multi‐omics	DAVID	https://david.ncifcrf.gov/home.jsp	The Database for Annotation, Visualization and Integrated Discovery (DAVID) provides a comprehensive set of functional annotation tools for investigators to understand the biological meaning behind large lists of genes. These tools are powered by the comprehensive DAVID Knowledgebase built upon the DAVID Gene concept which pulls together multiple sources of functional annotations
Genomics, Transcriptomics, Epigenomics	GeneCodis	https://genecodis.genyo.es/	Gene annotations co‐occurrence discovery
Genomics, Transcriptomics, Multi‐omics	GO	https://www.geneontology.org/	The Gene Ontology (GO) knowledgebase is the world's largest source of information on the functions of genes. This knowledge is both human‐readable and machine‐readable, and is a foundation for computational analysis of large‐scale molecular biology and genetics experiments in biomedical research
Genomics, Transcriptomics, Metabolomics, Multi‐omics	KEGG	https://www.kegg.jp/	A database resource for understanding high‐level functions and utilities of the biological system, such as the cell, the organism, and the ecosystem, from molecular‐level information, especially large‐scale molecular datasets generated by genome sequencing and other high‐throughput experimental technologies
Genomics, Transcriptomics, Multi‐omics	GSEA	https://www.gsea‐msigdb.org/gsea/index.jsp	Gene Set Enrichment Analysis (GSEA) is a computational method that determines whether an a priori defined set of genes shows statistically significant, concordant differences between two biological states (e.g., phenotypes)
Transcriptomics	miRBase	https://www.mirbase.org	microRNA database, the archive for microRNA sequences and annotations
Transcriptomics	Tools4miRs	https://tools4mirs.org	The first, manually curated platform gathering at the present over 170 methods for the broadly defined miRNA analysis. All tools in Tools4miRs are classified into four general and seven more detailed categories
Transcriptomics	miRPathDB	https://mpd.bioinf.uni‐sb.de/	With its new custom‐tailored features is one of the most comprehensive and advanced resources for miRNAs and their target pathways
Transcriptomics	HMDD	http://www.cuilab.cn/hmdd	A database that curated experiment‐supported evidence for human microRNA (miRNA) and disease associations
Transcriptomics	MISIM	http://www.lirmed.com/misim/Home	Based on miRNA‐disease data from the HMDD v3.0 database and the miRNA‐disease data were curated using miRBase(Release 22.1), and can offer the miRNA functional similarity in more details (e.g., stratifying by up and down‐miRNAs), higher quality, and more accurate enrichment analysis to users
Transcriptomics	OncomiR	http://www.oncomir.org/	An online resource for exploring miRNA dysregulation in cancer. (1) The identification of cancer‐relevant miRNAs; (2) De novo analysis based on miRNA expression
Transcriptomics	miRNACancerMap	http://cis.hku.hk/miRNACancerMAP/	A user‐friendly web server with integrated data sources and computational workflow, allowing users to search for miRNA‐cancer information, and easily analyze their own miRNA data by multiple miRNA algorithms with extensive annotation, under the framework of network topology characterization and interactive interrogation
Transcriptomics	DIANA TOOLS	https://dianalab.e‐ce.uth.gr/html/dianauniverse/	Aim of DIANA Tools is to provide algorithms, databases, and software for interpreting and archiving data in a systematic framework ranging from the analysis of expression regulation from deep sequencing data, the annotation of miRNA regulatory elements and targets to the interpretation of the role of ncRNAs in various diseases and pathways, include (1) MicroRNA target prediction: microT‐CDS, microT v4; (2) Database of experimentally supported microRNA targets: TarBase v7.0; (3) Incorporating microRNAs in pathways: mirPath; (4) Analysis of expression data for microRNA function: DIANA‐mirExTra; (5) DIANA‐web server 5.0
Genomic, Transcriptomics	miRWalk	http://mirwalk.umm.uni‐heidelberg.de/	miRWalk stores predicted data obtained with a machine learning algorithm including experimentally verified miRNA‐target interactions. The focus lies on accuracy, simplicity, user‐friendly design, and mostly up‐to‐date information
Genomic, Transcriptomics	miRDB	https://mirdb.org/	An online database for miRNA‐target prediction and functional annotations. All the targets in miRDB were predicted by a bioinformatics tool, MirTarget, which was developed by analyzing thousands of miRNA‐target interactions from high‐throughput sequencing experiments
Genomic, Transcriptomics	TargetScanHuman	https://www.targetscan.org/vert_80/	Search for predicted microRNA targets in mammals
Transcriptomics	NONCODE	http://www.noncode.org/	An integrated knowledge database dedicated to noncoding RNAs (excluding tRNAs and rRNAs). Now, there are 39 species in NONCODE including 16 animals and 23 plants. The source of NONCODE includes literature and other public databases
Transcriptomics	RNAcentral	https://rnacentral.org/	The noncoding RNA sequence database, a comprehensive ncRNA sequence collection representing all ncRNA types from a broad range of organisms
Transcriptomics	Lnc2Meth	http://bio‐bigdata.hrbmu.edu.cn/Lnc2Meth/	Aim to provide a comprehensive resource and web tool for clarifying the regulatory relationships between human lncRNAs and associated DNA methylation in diverse diseases
Transcriptomics	LncRBase	http://dibresources.jcbose.ac.in/zhumur/lncrbase2/start2.php	To provide information on basic lncRNA transcript features, with additional details on genomic location, overlapping small noncoding RNAs, associated Repeat Elements, lncRNA promoter information, etc. with an added section of clinical importance named “ClinicLSNP” which hosts information about SNPs present within lncRNAs
Transcriptomics	LncRNASNP2	http://bioinfo.life.hust.edu.cn/lncRNASNP#!/	A database providing comprehensive resources of single‐nucleotide polymorphisms (SNPs) in human/mouse lncRNAs. It contains SNPs in lncRNAs, SNP effects on lncRNA structure, mutation in lncRNAs, and lncRNA:miRNA binding
Transcriptomics	circBase	http://www.circbase.org/	To explore public circRNA datasets and download the custom Python scripts needed to discover circRNAs in your own (ribominus) RNA‐seq data
Transcriptomics	circbank	http://www.circbank.cn/	A comprehensive database of human circRNA which included more than 140,000 human annotated circRNA from different sources. A novel nomenclature system based on the host gene name, start position, and end position was applied for the naming of circRNA
Transcriptomics	CircFunBase	http://bis.zju.edu.cn/CircFunBase/	A web‐accessible database that aims to provide a high‐quality functional circRNA resource including experimentally validated and computationally predicted functions. The current version of CircFunBase documents more than 7000 manually curated functional circRNA entries, mainly including Homo sapiens, Mus musculus, etc. CircFunBase provides visualized circRNA‐miRNA interaction networks
Transcriptomics	TSCD	http://gb.whu.edu.cn/TSCD/	This database is constructed to provide a global view of tissue‐specific circRNA in main tissues of human and mouse and is able to contribute to identify new markers for organogenesis and development disease
Transcriptomics	CSCD	http://gb.whu.edu.cn/CSCD/	A database developed for cancer‐specific circRNAs. CSCD collected available RNA sequencing (total RNA with rRNA depleted or polyA‐enriched) datasets from 87 cancer cell line samples
Genomic, Transcriptomics	ENCORI/starBase	https://rnasysu.com/encori/	An openly licensed and state‐of‐the‐art platform to facilitate the integrative, interactive, and versatile display of, as well as the comprehensive annotation and discovery of, RNA–RNA and protein–RNA interactions by developing new software and pipelines to deeply mining thousands of high‐throughput sequencing data of RNA–RNA interactome, CLIP‐seq, and degradome‐seq
Genomics, Transcriptomics, Epigenomics, Proteomics, Multi‐omics	Expasy	https://www.expasy.org/	It is an extensible and integrative portal which provides access to over 160 databases and software tools, developed by SIB Groups and supporting a range of life science and clinical research domains, from genomics, proteomics, and structural biology, to evolution and phylogeny, systems biology, and medical chemistry
Proteomics	AACompIdent	https://bio.tools/aacompident#!	Protein identification by amino acid composition, and optionally pI, Mw, species, UniProtKB keyword, and calibration protein. Several constellations are available, corresponding to various amino acid analysis techniques
Proteomics	UniProt	https://www.uniprot.org/	The world's leading high‐quality, comprehensive, and freely accessible resource of protein sequence and functional information
Genomics, Proteomics	SMART	http://smart.embl‐heidelberg.de/	Two different modes: normal or genomic. The main difference is in the underlying protein database used
Genomics, Transcriptomics, Proteomics	CR2Cancer	http://cis.hku.hk/CR2Cancer/	A comprehensive annotation and visualization database for CRs in human cancer constructed by high‐throughput data analysis (e.g., TCGA and CCLE) and literature mining (PubMed)
Proteomics	COBALT	https://www.ncbi.nlm.nih.gov/tools/cobalt/re_cobalt.cgi	Computing a multiple protein sequence alignment using conserved domain and local sequence similarity information
Genomics, Proteomics	Clustal Omega	https://www.ebi.ac.uk/Tools/msa/clustalo/	A new multiple‐sequence alignment program that uses seeded guide trees and HMM profile–profile techniques to generate alignments between three or more sequences
Genomics, Proteomics	MultAlin	http://multalin.toulouse.inra.fr/multalin/	Multiple‐sequence alignment by Florence Corpet
Proteomics	InterPro	http://www.ebi.ac.uk/interpro/	Providing functional analysis of proteins by classifying them into families and predicting domains and important sites
Proteomics	ELM	http://elm.eu.org/	This computational biology resource mainly focuses on annotation and detection of eukaryotic linear motifs (ELMs) by providing both a repository of annotated motif data and an exploratory tool for motif prediction
Proteomics	PROSITE	https://prosite.expasy.org/	Database of protein domains, families, and functional sites
Proteomics	NLSdb	https://rostlab.org/services/nlsdb/	Providing multiple services concerning nuclear export and localization signals (NES/NLS)
Transcriptomics, Proteomics	Proteinatlas	https://www.proteinatlas.org	A Swedish‐based program initiated in 2003 with the aim to map all the human proteins in cells, tissues, and organs using an integration of various omics technologies, including antibody‐based imaging, mass spectrometry‐based proteomics, transcriptomics, and systems biology
Proteomics	RCSB PDB	https://www.rcsb.org	The US data center for the global Protein Data Bank (PDB) archive of 3D structure data for large biological molecules (proteins, DNA, and RNA) essential for research and education in fundamental biology, health, energy, and biotechnology
Proteomics	Binding DB	https://www.bindingdb.org/bind/index.jsp	Aiming to make experimental data on the noncovalent association of molecules in solution searchable via the WWW. The initial focus is on biomolecular systems, but data on host–guest and supramolecular systems are also important and being included over time
Genomics, Proteomics	String	https://string‐db.org	A Core Data Resource as designated by Global Biodata Coalition and ELIXIR
Metabolomics, Multi‐omics	SBML	https://sbml.org/	A free and open data format for computational systems biology that is used by thousands of people worldwide
Transcriptomics. Epigenomics, Metabolomics, Multi‐omics	SysBio.se	https://www.sysbio.se/	Systems and Synthetic Biology is headed by Professor Ivan Mijakovic and is composed of several labs directed by faculty members at the department. The lab and computational facilities are shared among all members of Systems and Synthetic Biology
Metabolomics, Multi‐omics	Gaggle	http://gaggle.systemsbiology.net	An open‐source software system for integrating bioinformatics software and data sources

## 4 CONCLUSION AND FUTURE PROSPECT

**FIGURE 3:**
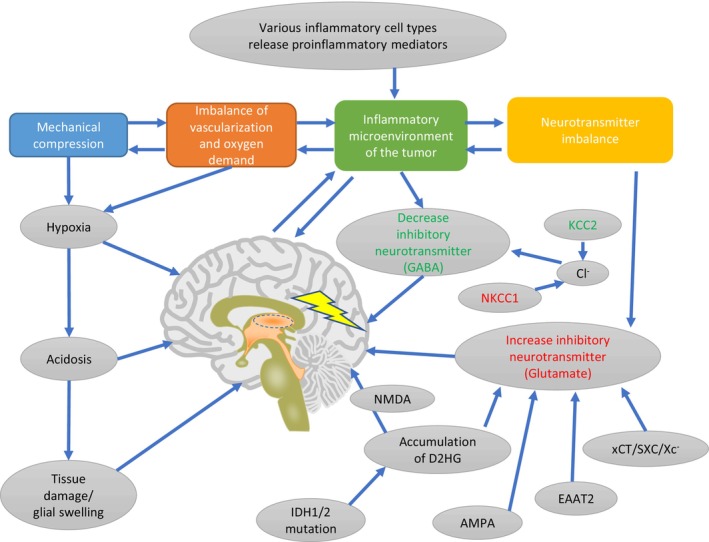
A brief framework of the pathogenic mechanisms involved in brain tumor‐related epilepsy.

The generation of BTRE is a complex, multifactor interaction pathophysiology process, involving numerous metabolic changes and pathway changes. In this study, we conducted a systematic review of molecular biomarkers associated with BTRE. We summarized the roles of molecules involved in immune responses, synaptic transmission, and cell cycle control in the development of epilepsy among patients with brain tumors from the perspective of omics (Figure 3).

Compared with a large number of omics studies on tumor tissues, there is a lack of omics studies on peripheral tumor tissues, which is a pity for current research. Epileptic activity is originated from peritumor regions found within 1–2 mm of the tumor border rather than the tumor itself.^156^ In the current systematic review, only a limited number of studies compared peritumoral tissues with tumor tissues or normal brain cells. Therefore, future studies with larger sample sizes are necessary to investigate the correlation between pathological characteristics of peritumoral tissues and seizures in glioma patients.

According to recent studies, antiepileptic medications like valproic acid and levetiracetam extended the longevity of glioma patients, whereas alkylating treatments like temozolomide reduced the frequency of seizures.^157^, ^158^ The research on antiepileptic mechanism and antitumor mechanism of many drugs can promote each other, and both can improve our understanding of the mechanisms by which brain tumors trigger seizures. There are several new omics techniques available, but very few have been used to study tumor‐associated epilepsy to date. For example, it is well known that tumor is a heterogeneous mass of cells, and slightly different tumor cells may play different roles in the generation of epileptic species. The results obtained by today's common omics techniques are averages of these cells, and the differences have not been studied well. In recent years, the rapid development of single‐cell detection technology and spatial omics technology should play an important role in this respect. We expect to see more and more in‐depth studies of tumor‐associated epilepsy based on these new omics techniques in the future, as the cost decreases the availability of these new technologies.

In order to more accurately, comprehensively, and systematically understanding the pathophysiological mechanism, omics study in BTRE will be widely performed. Screening the significantly related genes, proteins, metabolites, and metabolic pathways, afterward providing a basis for subsequent functional verification. Furthermore, clinical application of omics biomarkers is still in the early stages, and there will be more opportunities in the future.

## AUTHOR CONTRIBUTIONS

*Methodology*: YD, DF, BZ, ZL. *Writing – original draft*: YD, RL, BZ. *Writing – review & editing*: YD, RL, DF, YS, LC. *Investigation*: AC, RC. *Supervision*: YD, BC, WZ, LC. *Funding acquisition*: YD, ZW. *Project administration*: LC. All authors read and approved the final manuscript.

## FUNDING INFORMATION

This work was supported by National Natural Science Foundation of China (Grant No. 82202605, 82274122, 81772664), Zhejiang Provincial Natural Science Foundation of China (Grant No. LQ21H200007), Hangzhou Medical College Fundamental Scientific Research Project of China (Grant No. KYQN202116), and Zhejiang Provincial Medical and Health Technology Project of China (Grant No. 2024KY020, 2023KY519, 2022KY507).

## CONFLICT OF INTEREST STATEMENT

The authors declare no conflict of interest.

## Supporting information


Table S1.

## Data Availability

The data relevant to the review article are within the manuscript.
